# A Case of Pneumonia with Cerebral Complications

**Published:** 1898-02-19

**Authors:** Sidney Coupland

**Affiliations:** Physician to the Hospital


### Medical Progress and Hospital Clinics.

[*The Editor will be glad to receive offers of co-operation and contributions from members of the profession. All letters should be addressed to* The Editor, at the Office, 28 & 29, Southampton Street, Strand, London, W.C.]

### A CASE OF PNEUMONIA WITH CEREBRAL COMPLICATIONS.

A Clinical Lecture given at the Middlesex Hospital by Sidney Coupland, M.D., F.R.C.P., Physician to the Hospital.

(*Continued from page* 341.)

That delirium was the forerunner of the condition which gives such interest to this case. It recurred in the evening with much severity, passing into a state bordering on mania, in which he was not only noisy but even dangerously violent, so that he had to be removed to a special ward, and during Monday was placed in the padded room, and encased in the "camisole," the garment which has been beneficently substituted for the old strait-jacket of unhappy memory. He was thus placed out of means of injuring either himself or others, and in my absence from town he was given, on Dr. Cayley's advice, a hypodermic injection of hyoscyine, which had the effect of procuring sleep, from which he awoke much calmer. Thus the whole character of his illness had changed. The pulmonary condition was lost sight of in the overwhelming severity of this acute mental derangement.

Observe now that the disease had reached its crisis. The pyrexia abated, the temperature falling to 100 deg. by the Tuesday morning, and continuing to fall uninterruptedly during the next 36 hours, when it reached 98 deg.

Meanwhile, however, his mental condition did not improve. It had passed beyond the brief outburst of maniacal excitement, and entered on a condition to which the term "melancholia" would be more appropriate. He was quiet, sullen, stubborn, and suspicious, strenuously and successfully resisting attempts to give him food, imagining that his attendant wished to poison him. So that all through Tuesday he had no food, until, at ten p.m., Mr. Ward fed him forcibly by means of the stomach tube. He had been very wakeful all day, but some sleep was procured at night by the hypodermic injection of morphia, strychnia, and atropia.

On the next morning he was at first still averse to taking food; but this difficulty soon passed, and as the day wore on his reason returned, and he was enabled to be taken out of the padded room, and replaced in the special ward. He now slept well and gave us no more anxiety, so that on the 22nd he was brought back to Hertford.

You will see from the chart that the morning temperatures were now subnormal, and the pulse had fallen to 84 deg. Examination of the chest made from time to time showed that the lung was slowly clearing up; coarse redux crepitation appearing, and the area of dulness becoming more restricted. But even now—ten days after the crisis—there is still dulness over the lower two-thirds of the lower lobe of the lung.

His convalescence has been characterised by a curious phenomenon, for which I have no ready explanation. I refer to the passage of blood with the motions. I can only conjecture that this came from the large bowel, but whether from a polypus or from an undetected hæmorrhoid I cannot speak positively. After continuing for two or three days, during which he was put on milk diet, it has entirely ceased.

And now permit me to make a few remarks upon the special feature of this case—the outburst of maniacal delirium on the seventh day of the illness. Delirium (and I am not here speaking of delirium tremens) is a common symptom of acute pneumonia, just as it is of other fevers. It is often associated with headache, and is pronounced from the outset, so much so that the underlying disease is actually overlooked. Such delirium may be associated with the high temperature that characterises this disease, but this is not essential; and now that we know that pneumonia is not a mere localised affection of the lung, but a specific fever, it is probable that the action of the pneumonic toxine plays as great a part in the production of this and other cerebral symptoms as does the mere fact of pyrexia. In the present case, however, the onset of the disease, although characteristically sudden, was unaccompanied by any such cerebral disturbance. It was not until the time for the crisis was approaching that this happened, and had it actually coincided with the crisis its occurence might have been more explicable. For the perturbation of the whole economy by the sudden change which occurs at the crisis of a fever may, and does, in susceptible subjects, produce actual mania, and there is no reason why it should not be accompanied by a more transitory disturbance of the mental balance. But here the commencement of the cerebral excitement took place more than two days before the critical fall of temperature, although it is true there was a fall on the morning of the day following the first delirious night. In this respect, then, the case is exceptional. It is exceptional also in the brevity of the mental disorder, which rapidly passed through a cycle that may be described under the terms delirium, mania, melancholia, without implying that the subject should be regarded as an insane person. Yet he was to all intents "temporarily insane," and he may congratulate himself that the condition was not more prolonged. Sir S. Wilks says: "Delirium, in a general sense, implies disorder of the mind, and, according to this definition, the term is equivalent to insanity. The expression is used, however, in a much more limited sense, being made to signify that temporary disturbance which occurs in connection with other diseases, generally of a febrile kind. . . ." And again: ". . . the delirium of febrile diseases must not be confounded with the mental disturbance which is occasionally met with in those affections after the acute symptoms have passed. Ordinary delirium occurs at the very beginning of the fever, whereas this subsequent disturbance takes place when the fever is over. . . . The patient has, perhaps, no febrile delirium, or very little, but after the subsidence of this, as well as the other acute symptoms, he becomes maniacal in the ordinary sense of the term, talking incoherently, not knowing his friends, and acting in every way as a lunatic. It may be a question whether this maniacal state, as well as the degree of febrile delirium, arises from any predisposition to mental disturbance. . . ." (Tuke's "Dict. Psych. Med."; art., "Delirium.")

(*To be continued.*)

